# Healing Through Empowerment and Active Listening: Experience‐Based Co‐Design of a Nurse‐Led Personalised Self‐Care Support Intervention for Primary Care Patients With Diabetic Foot Ulcers

**DOI:** 10.1111/hex.70386

**Published:** 2025-08-23

**Authors:** Xiaoli Zhu, Eng Sing Lee, Frederick H. F. Chan, Ruoyu Yin, Rachel W. S. Koh, Phoebe X. H. Lim, Carpenter Judith, Voon Hooi Lim, Richard S. Y. Low, Yee Chui Chen, Yan Chen, Xiuhong Wang, Pei Pei Ng, Catherine T. Y. Tan, Sonia Tan, Katrina Pereira, Konstadina Griva

**Affiliations:** ^1^ NHG Polyclinics, NHG Health Singapore; ^2^ Nanyang Technological University Lee Kong Chian School of Medicine Singapore; ^3^ Department of Social Work and Social Administration The University of Hong Kong Hong Kong SAR China; ^4^ University Hospitals of Derby & Burton UK

**Keywords:** co‐design, diabetic foot ulcer, empowerment, personalised, primary care, self‐care

## Abstract

**Background:**

The rising prevalence of diabetic foot ulcers (DFUs) highlights the need for effective self‐care interventions. Despite strong evidence supporting their effectiveness, patient engagement, uptake, and integration into routine care remain limited. Co‐design approaches can enhance the relevance and adoption of interventions but are underutilized in DFU management.

**Objectives:**

This study outlines the development of Healing through Empowerment and Active Listening (HEALing), a self‐care intervention for patients with DFU, grounded in Self‐Determination Theory and Motivational Interviewing, and developed using an experience‐based co‐design approach.

**Design, Setting and Participants:**

The 27‐month co‐design process consisted of three phases involving patients, caregivers, and healthcare professionals (HCPs) from across a primary care cluster in Singapore. Phase 1 (16 months) included qualitative interviews with patients (*N* = 27), caregivers (*N* = 5), and HCPs (*N* = 8), analyzed via reflexive thematic analysis, alongside a quantitative survey (*N* = 186), analyzed using structural equation modelling to identify intervention determinants. Phase 2 (9 months) involved co‐design workshops with patients (*N* = 10) and wound care nurses (*N* = 6) to define the intervention's content and delivery approach. In Phase 3 (2 months), iterative meetings refined the intervention's procedures, tools, and materials. Qualitative data from Phases 2 and 3 were thematically analyzed.

**Results:**

Key barriers to DFU self‐care included limited control over ulceration and treatment, low confidence, negative emotions, and misperceptions about the condition. Personalised care and clinician‐facilitated motivation emerged as key enablers. HEALing targets five core self‐care components: (1) wound care, (2) foot care and footwear, (3) diabetes care, (4) treatment seeking, and (5) managing worries and concerns. The personalised components are delivered using a card‐sorting tool during clinic‐integrated sessions by trained wound care nurses. The HEALing delivery pathway comprises three 30‐min sessions at 2‐week intervals. During workshops, participants perceived HEALing as supporting patient‐ and clinician‐endorsed person‐centred care and collaborative planning, while also identifying potential implementation challenges, including training needs and structural barriers.

**Conclusions:**

HEALing positions patients as experts by experience, addressing the psychological and behavioural complexities of DFU care. This nurse‐led and stakeholder‐endorsed intervention is currently undergoing feasibility evaluation.

**Patient and Public Involvement:**

Individuals with DFUs, their caregivers, and those in with post‐healing remission, and wound care nurses contributed to the co‐design of HEALing by identifying intervention targets and informing the intervention's content and delivery.

**Abbreviations**
DEFINITE: Care programme (Diabetic Foot in Primary and Tertiary Care)
DFU: diabetic foot ulcer
EBCD: experience‐based co‐design
GUIDED: guidance for reporting intervention development
HCP: healthcare professional
HEALing: healing through empowerment and active listening
MI: motivational interviewing
SDT: self‐determination theory
TIDieR: template for intervention description and replication

## 1 Introduction

The increasing prevalence of diabetic foot ulcers (DFUs) presents a major challenge in diabetes care, which demands innovative, patient‐centred interventions. DFUs are a severe complication of diabetes mellitus that affects more than 18.6 million individuals annually and affects nearly 500 million adults worldwide [1]. They are characterised by prolonged healing, a 40% recurrence rate within 1 year [2], and a 20% risk of lower extremity amputation [3]. DFUs also impose significant psychosocial burdens, including depression, anxiety, and social isolation [4, 5, 6]. Additionally, they contribute up to 30% of diabetes‐related healthcare costs [7].

Effective DFU management depends on sustained self‐care, including wound care, glycemic control, foot care (i.e., self‐inspection, skin care), therapeutic footwear use, and adherence to multidisciplinary team (MDT) recommendations [8, 9, 10]. However, major gaps persist: over 50% of patients with DFU engage in inadequate self‐care practices [11]; up to 50% do not adhere to wound and MDT visits [12]; fewer than 20% receive recommended foot screenings; and up to 65% have HbA1c > 53 mmol/mol (7%) [13]. Despite its critical role in prognosis, self‐care in DFU management remains underemphasised.

While patient behaviour is crucial for positive clinical outcomes, multiple barriers hinder self‐care adherence, including negative beliefs about DFUs, emotional distress, and the complexity of self‐care routines, which often leave patients feeling overwhelmed and ill‐equipped to manage their condition effectively [5, 14, 15]. Additionally, poor patient–provider communication weakens trust, limits informed decision‐making, and further reduces motivation for self‐care [5, 15]. These challenges create a cycle of disengagement and disempowerment, ultimately compromising DFU outcomes. Patients consistently highlight the need for supportive, empathetic, nonjudgmental healthcare interactions that foster trust and encourage long‐term self‐care engagement [5, 15, 16].

To date, numerous interventions for DFU management have been developed, but most have focused on educating patients to increase their knowledge and skills in DFU care [17, 18, 19], which, despite being effective in improving self‐care behaviours, overlooks other key barriers to adherence, such as emotional distress and a lack of confidence. A psychoeducational intervention combining patient and family education with nurse‐led psychosocial care resulted in reductions in anxiety and depression related to DFU, yet self‐care was not assessed as an outcome [20]. A comprehensive intervention addressing both psychosocial and behavioural factors is needed to ensure sustainable self‐care in DFU management.

The Self‐Determination Theory (SDT) provides a valuable theoretical foundation to guide the development of self‐care support interventions in DFU care. SDT posits that individuals have three fundamental psychological needs that drive behaviour: autonomy (a sense of choice), competence (confidence in achieving outcomes), and relatedness (feeling socially accepted) [21]. SDT has been widely applied in healthcare research, with evidence indicating that greater psychological need support and autonomous motivation are associated with positive changes in health behaviours [22]. Interventions that incorporate the satisfaction of these psychological needs are more likely to facilitate the internalisation and maintenance of health behaviours over time [23, 24].

While SDT outlines the mechanisms underpinning motivation and self‐regulation, Motivational Interviewing (MI) serves as a practical, patient‐centred counselling approach to operationalise these principles [24, 25]. MI strengthens autonomy through patient‐directed goal‐setting rather than prescriptive advice, enhances competence by collaboratively exploring and addressing barriers to build self‐efficacy, and fosters relatedness through empathetic, non‐judgemental communication that strengthens trust between patients and healthcare providers [23, 24, 26]. MI has demonstrated effectiveness in primary care and diabetes management, enhancing behavioural change, emotional adjustment, and long‐term self‐management adherence [27, 28, 29, 30, 31, 32]. In podiatry clinics, MI‐based interventions have shown promise in improving adherence to offloading and foot care behaviours [33]. However, despite the central role of nurses in wound management and foot care education [14, 34, 35], MI remains underutilised within nurse‐led DFU services in primary care settings.

Integrating MI within an SDT‐informed framework offers a theoretically grounded, evidence‐based approach [23, 24] to supporting DFU self‐care and psychological well‐being. This integration fosters autonomous motivation and psychological need satisfaction, providing a personalised and sustainable foundation [24, 31, 36] for intervention delivery within primary care.

Co‐design, a participatory method engaging patients, caregivers, and healthcare professionals (HCPs), ensures that interventions are innovative, adaptable, and equitable by fostering collective ownership and grounding development to real‐world needs [37, 38]. experience‐based co‐design (EBCD), a structured approach rooted in patient and staff experiences, uses storytelling to identify systemic inefficiencies and enhance service usability. Through its six‐step process—integrating interviews, collaborative workshops, and iterative reflection—EBCD engages service users, carers, and clinicians to drive meaningful change [37]. While EBCD has improved outcomes in diverse chronic conditions [37, 39, 40], its application to DFU care remains novel, offering untapped potential to address critical barriers such as fragmented care pathways and patient disengagement.

Despite the introduction of the Diabetic Foot in Primary and Tertiary (DEFINITE) Care initiative – a health system innovation to improve DFU care coordination in Singapore [41] – ongoing self‐care challenges, limited patient engagement, and non‐adherence continue to hinder outcomes [12]. While primary healthcare professionals are well positioned to deliver theoretically informed, evidence‐based self‐care support interventions [42], research on their application in the DFU population remains limited.

To address the critical gaps in DFU care, this study employed a collaborative EBCD approach to develop Healing through Empowerment and Active Listening (HEALing), a nurse‐led self‐care intervention integrated into routine wound care clinic visits. Rooted in SDT and MI, HEALing targets emotional distress, self‐care complexity, and strained patient – provider relationships by empowering individuals with DFUs through motivational communication with the support of psychological needs.

This paper details the co‐design process, demonstrating how stakeholder collaboration bridges the gap between theoretical frameworks, clinical practice, and patient priorities to transform DFU care.

## 2 Methods

### 2.1 Design

The intervention development process was informed by the Guidance on Complex Intervention Development, including seeing intervention development as a dynamic iterative process involving stakeholders, reviewing published research evidence, drawing on existing theories, articulating programme theory, undertaking primary data collection, understanding context, paying attention to future implementation in the real world, and designing and refining an intervention via iterative cycles of development with stakeholder input throughout [38].

In the current study, we structured the intervention development process into three phases. In phase 1, we conducted mixed methods studies (16 months) that included surveys and interviews with patients, caregivers, and healthcare professionals to map the needs, priorities, and drivers of DFU outcomes. In phase 2 (EBCD, 9 months), a series of co‐design workshops with stakeholders were undertaken to scope out and define the intervention's content and delivery methods. In phase 3 (2 months), iterative team meetings reviewed all inputs gathered from phase 2 and finalised all the intervention procedures, tools, materials, and intervention pathway.

**Figure 1:**
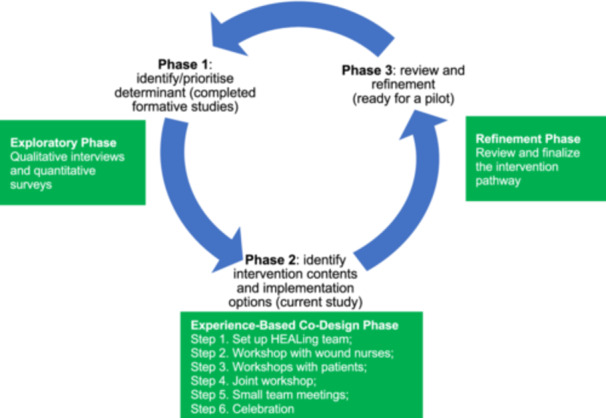
Intervention development process: The cycle includes three phases (inspired and adapted from the Guidance on How to Develop Complex Interventions to Improve Health and Healthcare Communication [38] and prior work in other populations. The green rectangular boxes indicate the phases and steps of the experience‐based co‐design cycle/process (adapted from prior co‐design work in other populations [37, 39, 40]. [Color figure can be viewed at wileyonlinelibrary.com]

Figure 1 illustrates the three intervention development phases, and Table 1 details the objectives, actions, methods, data types, and outputs of each phase. The current paper primarily focuses on Phases 2 and 3, with findings from Phase 1 reported elsewhere [5, 35, 44].

**Table 1:** Methodology of the co‐design process.

Co‐design process	Objectives and actions	Methods	Participants, data types and collection point	Outputs
Phase 1: exploratory phase (16 months) (May 2022–Aug 2023)	Identify barriers and enablers to DFU care using interviews and quantitative surveys	Interview and quantitative survey studies (completed and published) Analysis: reflexive thematic analysis and structural equation modelling	Patients with DFU (*N* = 201) and their caregivers (*N* = 55); patients with posthealing foot in remission (*N* = 12); HCPs (*N* = 8) Audio interviews and field notes were collected during individual interviews and focus group discussions. Data on patient‐reported outcome measures (PROM) related to psychological factors and self‐care behaviour were collected through quantitative surveys.	Identify needs and prioritise target determinants
Phase 2: EBCD phase (9 months)(Sep 2023– May 2024)	Scope intervention contents and implementation options through EBCD process, i.e., facilitate (joint) workshops, collect reflections, feedback on shared experiences, and understand contexts	Six steps of EBCD phase a. Set up HEALing team b. Workshop with wound care nurses c. Workshop with patients d. Joint workshop e. Small team meetings f. Celebration	HEALing team: Patients with DFU (*N* = 10), wound care nurses (*N* = 6), and academic researchers (*N* = 7) Audio records of workshop discussion, and story dialogue/storytelling on real‐world care scenarios, field notes, and co‐design meetings notes. Data were collected from a series of co‐design workshops and joint workshops with patient and wound care nurse participants.	List of intervention contents and delivery methods
		Workshops (see Additional file: S1 for HEALing workshop outlines) and iterative co‐design meetings including group discussions, role‐plays using deidentified real‐world vignettes drawn from patient‐facing encounters^a^, reflections, and observations. Key question guides used for facilitating a series of workshops are: For workshop with wound nurses: *What will help when giving information and providing knowledge and skills to support patient self‐care and wound healing, especially when your patients have low‐mood related to wound deterioration and slow healing*? For workshops with patients living with DFU: *What/How would you like your wound nurses to help you when giving information and providing knowledge and skills to support your self‐care and wound healing, especially when things did not go well, e.g., slow wound healing or wound deterioration, or when you felt being overwhelmed?* For joint workshop with both patient and wound nurse participants: *What would be your suggestions to improve the contents options and delivery methods? For example, potential challenges or top concerns – for both patient and wound care nurses; expectations from your wound care nurses with regard to self‐care support – for patients*. Analysis: audio recorded workshops were transcribed verbatim and analyzed thematically.		
Phase 3: refinement phase (2 months) (June 2024–July 2024)	Review, refine, and finalise all intervention contents, tools and materials used	Co‐design team meetings and group discussions. Analysis: meeting notes were analyzed thematically.	Wound care nurses (*N* = 6) and academic researchers (*N* = 7) Notes of recursive co‐design meetings including mock intervention role plays and reflections, story dialogue. Data were collected from a series of co‐design team meetings.	HEALing programme pathway

Abbreviations: EBCD, experience‐based co‐design; HCP, healthcare professional; HEALing: Healing diabetic foot ulcers through empowerment and active listening.

a Patient‐facing vignettes were used as discussion promots, as previously described [43], replacing trigger films that could not be created during Phase 1 interviews due to pandemic.

### 2.2 Setting

This study was conducted at nurse‐led wound care services within Singapore primary care polyclinics where the lead author had previously undertaken formative work [5, 35]. The nurse‐led wound care services provide wound care and education, caring for approximately 1400 incident DFU cases annually in collaboration with all healthcare professionals under the DEFINITE Care programme (Diabetic Foot in Primary and Tertiary Care) [41]. This multidisciplinary, interinstitutional initiative aims to reduce ulceration and amputation rates within a healthcare cluster in Singapore.

Primary care wound care nurses in the cluster play a central role in DFU care. They work with patients to establish wound care plans, perform regular dressings, and provide education on foot and wound self‐care every 3–5 days until wound closure, in close collaboration with primary care physicians to enhance healing and prevent complications.

### 2.3 Participants and Data Collection

Patients with DFU, family caregivers, and HCPs participated in the three‐phase intervention development process. Table 1 provides details on participant types, attendance across co‐design activities, data types, and collection points.

Data collection and recruitment details for Phase 1 are documented elsewhere [5, 35]. In Phase 2 and Phase 3, convenience sampling was employed to recruit participants for the EBCD workshops and co‐design meetings. Participants included (1) patients with DFUs receiving wound care at the participating primary care polyclinics and (2) wound care nurses with direct patient‐facing roles in DFU care across all polyclinics. Both groups were engaged as collaborative partners in the EBCD process. Wound care nurses were invited via email, and the lead author screened all potential participants for eligibility based on the inclusion criteria. All eligible individuals were asked to complete written informed consent forms.

### 2.4 Patient and Public Involvement

People with DFU as experts by experience and end‐users for HEALing were partners in the co‐design process. Their perspectives, feedback and experiences informed all phases – from the exploratory development and refinement of HEALing to its implementation and dissemination.

### 2.5 Programme Logic Model

**Figure 2:**
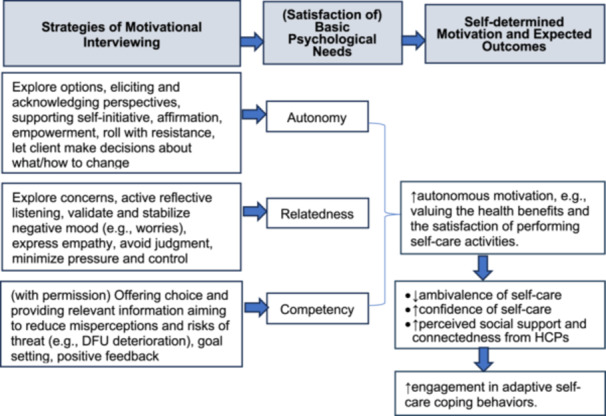
The HEALing logic model illustrates the complementary approaches of MI and SDT that can elicit adaptive behaviour change for better health outcomes and behavioural adoption, in which MI strategies provide support to satisfy the psychological needs for competence, autonomy, and relatedness. DFU, diabetic foot ulcer; HCP, healthcare professional; MI, motivational interviewing; SDT, self‐determination theory. [Color figure can be viewed at wileyonlinelibrary.com]

The study team developed a programme logic model to guide the design, delivery, and evaluation of the HEALing intervention, drawing on SDT, MI, relevant literature [21, 26, 33], and findings from Phase 1 [5, 35, 44] (Figure 2). The model delineates how specific MI techniques are intended to support the psychological needs for autonomy, competence, and relatedness, as articulated within SDT, to facilitate improved outcomes among individuals with diabetic foot ulcers.

Within the intervention, autonomy is supported by offering choices, providing meaningful rationales, acknowledging patient perspectives, and minimising external pressures [45, 46, 47]. Relatedness is addressed by demonstrating care, validating emotions, and providing unconditional positive regard, while competence is fostered through collaborative problem‐solving and the provision of appropriately challenging tasks [45, 46, 47]. The delivery of the intervention is informed by MI principles of partnership, acceptance, compassion, and empowerment, employing techniques such as open‐ended questions, affirmations, reflective listening, summarising, and structured information exchange to engage participants, explore concerns, resolve ambivalence, and enhance self‐care confidence [48, 49].

The programme logic model informs the structuring of intervention components, the preparation and training of intervention deliverers, and the evaluation of intervention processes and outcomes within the study.

Details of the logic model illustrating the theory of autonomy support based on SDT, including the proposed mechanism and targeted psychological/clinical needs of DFU care, are presented in Table 2.

**Table 2:** Logic model illustrating the theory of autonomy support based on self‐determination theory: Supporting diabetic foot ulcer self‐care and healing through empowerment and active listening (HEALing).

Identified Determinants	HEALing mechanisms: SDT‐based strategies and MI supported activities	Targeted basic psychological needs	Self‐determined motivation	*Targeted clinical/emotional care needs (intervention options)	Hypothesised outcomes
Lack of control over DFU and its treatment, low confidence in self‐care	Agenda mapping of DFU self‐care tasks to identify areas of competency (strengths) and areas in need of improvement; eliciting and acknowledging patient perspective about DFU self‐care and the recommended changes (*MI process: engaging; example of MI tools/strategies: affirmations)* Champion Autonomy and bolster competency – agenda mapping for patient to choose DFU self‐care topic; draw out motivation toward the DFU topic (*MI processes: focusing (agenda mapping); and evoking (e.g. with change focused open‐ended questions, and summary)* Collaborative advice given to nurture competency; using the Ask‐Offer‐Ask framework of MI (*MI process: evoking and planning*) Goal setting considering its benefits, barriers, and importance to practice (MI process: planning example MI tools: transitional summaries and Ask‐Offer‐Ask framework)	Autonomy (refers to the need to feel that one is acting out of a sense of volition and self‐endorsement) Competence (refers to the need to feel confident in achieving outcomes and effective in one's behaviour).	Valuing the satisfaction of patients' basic psychological needs and the health benefits	Content options: a. *Wound care* b. *Foot care and footwear* c. *Diabetes care* d. *Treatment seeking*	More adaptive and confident self‐care, e.g., wound self‐care; self‐care in foot and footwear; diabetes self‐care; treatment seeking
				*Delivery options* Individualised (1‐to‐1) using card‐sorting tool, clinic‐integration, by the same nurse	
Negative emotion (related to DM and DFU healing prognosis)	Showing empathy and acknowledging, validating (negative) feelings associated DFU (*MI process: engaging and focusing; MI spirit: acceptance and compassion)*, focusing on listening and nonjudgmental. Providing a warm positive interpersonal care and feedback, explore the patients' personal interest and motivation to perform self‐care (*MI process:* evoking) Acknowledging a patient's perspective, (with permission) offering meaningful choices with a rationale while remaining nonjudgmental about patient choices help foster a sense of relatedness (*MI process: planning Ask‐Offer‐Ask Framework and reflections; MI spirit: acceptance*)	Relatedness (refers to the need to be cared for and caring for others)	Valuing the satisfaction of patients' basic psychological needs and the health benefits	Content options: e. *My worries and concerns*	Positive psychological adjustment, e.g., adaptive coping
				*Delivery options* Individualised (1‐to‐1) using card‐sorting tool, clinic‐integration, by the same nurse	
Misperceptions of DM and DFU.	Elicit and understand patients views and misperception. MI spirit to guide the process: partnership, acceptance, compassion, empowerment. MI skills: open‐ended questions, reflections to show understanding. MI process (engage and evoke). Collaborative advice to address misperceptions; using the Ask‐Offer‐Ask framework of MI to offer alternative views on DFU or adaptive DFU with permission. (*MI process: evoking*) Help setting realistic goals (*MI process: planning*)	Autonomy Competence	Valuing the satisfaction of patients' basic psychological needs and the health benefits	Content options: a. *Wound care* b. *Foot care and footwear* c. *Diabetes care* d. *Treatment seeking*	More adaptive and competent self‐care, e.g., wound self‐care; self‐care in foot and footwear; diabetes self‐care
				*Delivery options* Individualised (1‐to‐1) using card‐sorting tool, clinic‐integration, by the same nurse	

*Note:* *Targeted clinical care needs (intervention options) were identified through thematic analysis of co‐design workshops, informing the content and delivery of the intervention (see Additional files: S4 and S6).

Abbreviations: DFU, diabetic foot ulcer; MI, motivational interviewing.

### 2.6 Data Analysis

In Phase 1, mixed‐methods studies were conducted to identify challenges, barriers, and determinants of DFU self‐care, including qualitative interviews with patients, family caregivers, and HCPs, as well as quantitative observational surveys to map the relationships between psychological and behavioural variables and foot self‐care. Further details are available in our previous publications [5, 35].

In Phases 2 and 3, co‐design workshops were audio‐recorded and transcribed verbatim. Anonymized transcripts, co‐design meeting notes, and researcher field notes were thematically coded and analyzed by ZX, PL, and RK. These analyses guided the refinement of the intervention contents and the prioritisation of key elements for intervention and implementation options.

### 2.7 Ethical Considerations

The studies were approved by the Domain Specific Review Board Singapore (Ref No. 2021/01074 for Phase 1; Ref No. 2022/00895 for Phase 2 and 3) and Institute Review Board Nanyang Technological University (NTU IRB‐2022‐338 for Phase 1; Ref No. IRB‐2023‐335 for Phase 2 and 3). All study participants across all phases provided written consent.

The co‐design process is reported in accordance with guidance for reporting intervention development studies in health research (GUIDED) [50] (Additional file: S2). We followed the template for intervention description and replication (TIDieR) [51] and provided guidance for better reporting of interventions (Additional file: S3).

## 3 Results

The demographic characteristics of study participants in Phases 2 and 3 are presented in Table 3, while details for Phase 1 participants are reported elsewhere [5, 35].

**Table 3:** Demographic characteristics of study participants in Phases 2 and 3.

Study ID	Gender	Role	Duration of DFU (month)	Years of experience of current role
P1	M	Patient participant with DFU	4	
P2	M	Patient participant with DFU	5	
P3	M	Patient participant with DFU	6	
P4	M	Patient participant with DFU	24	
P5	M	Patient participant with DFU	4	
P6	M	Patient participant with DFU	6	
P7	M	Patient participant with DFU	10	
P8	M	Patient participant with DFU	8	
P9	M	Patient participant with DFU	4	
P10	M	Patient participant with DFU	9	
N1	F	Wound care nurse participant		8
N2	F	Wound care nurse participant		8
N3	F	Wound care nurse participant		8
N4	F	Wound care nurse participant		6
N5	F	Wound care nurse participant		8
N6	F	Wound care nurse participant		3
R1	F	Associate Professor of Health Psychology and Behavioural Medicine/PhD supervisor		20
R2	M	Senior Consultant Family Physician/PhD supervisor		7
R3	F	PhD researcher		5
R4	F	Research coordinator		4
R5	F	Research assistant		4
R6	F	Research assistant		2
R7	F	Research assistant		2

**Figure 3:**
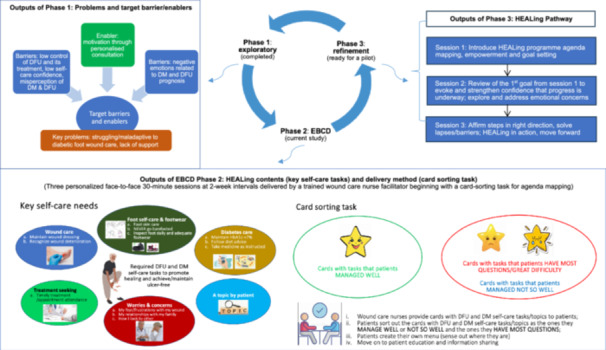
Overview of the co‐design process for HEALing intervention to support self‐care behaviours for primary care patients with diabetic foot ulcers, focusing on Phase 2 – experience‐based co‐design (EBCD). [Color figure can be viewed at wileyonlinelibrary.com]

The findings from HEALing development process are organised into three phases (with a focus on Phase 2 and 3), as outlined in Figure 1. The outputs of the intervention development process (Figure 3) include the prioritisation of key needs for intervention targets (outputs of Phase 1), intervention content and delivery options (outputs of Phase 2), and the HEALing pathways (outputs of Phase 3).

### 3.1 Phase 1: Identifying Challenges/Needs and Prioritising Determinants (Intervention Targets)

The experiences of DFU management were gathered through qualitative interviews with multiple stakeholders, including patients, family caregivers, and HCPs [5]. The understanding of the problem and the need for an intervention was informed by people with DFU as the experts by experience, their family caregivers and HCPs who are the end‐users of the intervention. A quantitative exploration further examined patients' beliefs about DFU and the factors influencing self‐care [35]. Triangulation of these findings revealed key determinants of effective self‐care, including lack of control over ulceration and its treatment, negative emotions associated with DFU and diabetes distress, low self‐care confidence, and misperceptions about diabetes and DFU (barriers) and motivation driven by personalised care supported by HCPs (an enabler). Details of qualitative and quantitative exploration from multiple stakeholders for potential barriers to and facilitators of their target behaviours are documented elsewhere [5, 35, 44]. These insights informed the framework development and guided the co‐design of the intervention.

### 3.2 Phase 2: Identify the Intervention Options From a Series of Workshops

#### 3.2.1 Content Options

Building on the prioritised determinants identified in Phase 1, Phase 2 employed a series of co‐design workshops with co‐design members (research participants; see Table 3) to identify and refine the intervention content for DFU self‐care needs. Initial workshops, conducted separately with wound care nurses and patients living with DFU, explored targeted clinical and emotional care needs and priorities. Subsequent joint workshops used storytelling and role‐playing with anonymised case examples to align and collaboratively refine the content.

This iterative process identified five non‐ranked core categories encompassing twelve subtopics: wound care (dressing maintenance, recognising wound deterioration); foot care and footwear (skin care, appropriate footwear, foot self‐inspection); diabetes care (glycaemic control, dietary adherence, medication adherence); treatment seeking (timely care and appointment attendance); and emotional concerns (fears about the wound, how I look to others, family relationships). Prioritised through participant feedback and lived experiences, these categories defined the targeted clinical and emotional self‐care needs for the intervention, addressing essential self‐care knowledge, care engagement, and psychological concerns, and emphasising the need for both practical and emotional support. A detailed summary of the themes for content options, supported by illustrative participant quotes, is provided in Additional file: S4.

To support patient autonomy and avoid perceived coercion, a sixth category, “a topic by patient,” was added. This element enables patients to prioritise their own concerns and motivations for behaviour change, ensuring that the intervention remains patient‐centred and adaptable to individual needs within real‐world clinical contexts.

Figure 3 (Phase 2 outputs) visualises these six categories as colour‐coded “circles”, each representing the twelve subtopics indication twelve self‐care needs that structure the HEALing sessions.

#### 3.2.2 Delivery Methods

Intervention content options and delivery methods were explored concurrently during the iterative co‐design workshops. To facilitate collaborative agenda mapping within sessions, a research team content expert proposed using a card‐sorting task to help patients identify their strengths and support needs in diabetes and DFU self‐care (Additional file: S5).

Using the card‐sorting tool to structure discussion, participants articulated preferences regarding *how*, *when*, and *by whom* the intervention should be delivered, which were further refined in small group meetings. Key delivery preferences included: (1) individualised one‐to‐one 30‐min sessions (*how*), (2) integration into routine clinic visits (*when*), and (3) delivery by the same wound care nurse to ensure continuity and trust (*who*).

Both patients and wound care nurses preferred personalised sessions delivered within routine clinics immediately after wound care procedures. This approach aligns with Phase 1 enablers – personalised care and clinician‐facilitated motivation – supporting feasibility and patient engagement. Participants recommended spacing sessions up to 2 weeks apart to allow adequate time for reflection and internalisation of behavioural changes.

A summary of these delivery preferences, with illustrative quotes from patients and wound care nurses, is provided in Additional file: S6. Grounded in participant insights, the intervention emphasises patient autonomy, flexible session structures, and collaborative goal setting between patients and wound care nurses to ensure care is tailored to individual needs and preferences. A group gathering was held at the end of this stage to celebrate the completion of the co‐design process.

Figure 3 (Phase 2 outputs) visualises the card‐sorting chart used during agenda mapping, illustrating the categories “*managed well*” and “*managed not so well*.”

### 3.3 Phase 3: HEALing Programme Pathway

In Phase 3, the intervention was refined based on outputs from Phases 1 and 2 through consecutive and iterative meetings with wound care nurse co‐designers. Their contributions ensured clinical feasibility and contextual relevance, focusing on the integration of the HEALing pathway into routine clinic workflows and resolving logistical considerations. These sessions continued until revisions reached a point of saturation, with minimal further changes required.

The refined intervention prototype comprises three structured, personalised face‐to‐face sessions, each lasting approximately 30 min. These are embedded into routine wound clinic visits and facilitated by the patient's wound care nurse at intervals of up to 2 weeks. Building on the content and delivery structure established in Phase 2, the HEALing pathway was organised into three progressive sessions. The first session introduces the programme through agenda mapping, empowerment strategies, and collaborative goal setting. The second session reviews progress toward the initial goal, explores emotional concerns, and reinforces self‐efficacy. The final session affirms progress, addresses lapses or barriers, and supports sustained behaviour change through collaborative action planning.

Figure 3 provides an overview of the co‐design process and illustrates key features of the HEALing sessions. Full workshop materials are available in Additional file: S1. In line with best practice guidance for complex intervention development [38], the HEALing intervention will continue to evolve through feasibility testing, evaluation, and implementation.

### 3.4 Perceptions of the Intervention and Implementation Challenges

Analysis of the co‐design workshops and meetings identified four key themes across micro (individual), meso (relational), and macro (system) levels. At the micro level, patients and clinicians viewed HEALing as a “patient‐ and clinician‐endorsed person‐centred care support” and highlighted patients' psychological adjustment needs and clinicians' training needs to better support these. At the meso level, participants emphasised the need for a “collaborative care plan” between patients and practitioners to support psychological adjustment. At the macro level, “structural barriers” were identified as implementation challenges, including financial constraints for patients and resource limitations for clinicians. Table 4 presents these themes alongside illustrative quotes from patients and wound care nurses, highlighting shared perspectives on implementation challenges and the intervention's perceived impact. Overall, both groups showed consistent alignment in their perceptions, priorities, and concerns, reinforcing HEALing's relevance and feasibility in real‐world settings.

**Table 4:** Themes and illustrative quotes from patients and wound care nurses on perceptions of the intervention and implementation challenges identified during co‐design workshops.

Temes/Perceptions, challenges	Perceived by patients (illustrative quotes)	Perceived by wound care nurses (illustrative quotes)
Theme 1: A patient‐ and clinician‐endorsed person‐centred care approach (***micro level***)	*It is a very good programme, as on one hand is what we do, how the nurses support us to do the things. (P5)* *Every one of us got our own experience of how we got into this situation. Each one of us are different when we came into this situation. I think we need to learn how to accept. It is an ongoing challenge, but I think we have a good, personalised care collaboration. (P3)*	*“Comprehensive list for self‐care tasks, a highly motivating and beneficial patient‐centred care programme” (N1)* *This programme combines both education and emotional support, which are crucial elements in effectively managing chronic conditions like diabetic foot ulcers. It is likely to have a positive impact on patient outcomes and satisfaction with their care (N2)*.
Theme 2: A collaborative care plan to support psychological needs (autonomy, connectedness and competence) *(**meso level**)*	*For us diabetic patients, it's good to have the connection with clinicians. What you're doing now, even if the wound care nurses have connections with the patients, I tell you the patients will respond to you, tend to listen more. I think this is the most important thing for patient care as our voice counts (P6)*. *It's tough to be alone in this diabetic journey. The programme is beneficial helping me feel that “I am not alone”, yea will never work alone now (P5)* *If this whole HEALing education programme has this kind of one‐to‐one, overall it's a good thing. Because I come here before, I never had this kind of great interpersonal discussion, so I think it's very good (P2)*	*A collaborative approach to supporting patients in managing their condition. Incorporating motivational interviewing techniques; can empower patients to take ownership of their health and adhere to necessary treatments and lifestyle changes (N2)*. *It is a holistic approach by emphasising the importance of addressing both physical and emotional aspects of healing, recognising that healing is not just about wound care but also supporting patients' overall well‐being and quality of life. Additionally, reinforcing the idea that healing is a journey that patients are not alone, providing ongoing support and follow up care to ensure patients feel supported and motivated to continue their progress (N3)*. *I like the part [card‐sorting], you ask the patient to choose topic for discussion, how much they are willing to do it after suggestion – such a nice two‐way collaborative communication. It gives patients autonomy to choose topics for discussion with their nurse. Then we can give them suggestions, customising to their needs, and check their understanding and acceptance. (N3)*
Theme 3: Training needs for wound care nurses and patient support (psychological adjustment) **(** ***micro level*** **)**	*We have been frustrating and feeling of being overwhelmed because of our conditions (P10)* *Unlike fever and sore throat, 2 to 3 days can recover, diabetic foot wounds take very long time to heal but still not healed after so long. Sometimes we are frustrated and losing hope. Yeah, we definitely need that kind of support (P5)*	*I think the essence of it is truly the way that we engage and empower, the way that we speak and how we built up our responses to positively adjust patients' responses. It truly matters on how we communicate with them and built up from there. However, very often I feel that we lack support to address this. I believe that structured training programme will build our confidence in this important area of patient care (N2)* *I think this red card [emotion feeling) here is very vital for both sides (nurses and patients). Because if we don't address this, the other cards [wound care, foot/footwear and diabetes care, treatment‐seeking] are not going to work, because the red cards are usually the main barrier. I think all of us agree, we truly want those because they have proven to work. However, they are not able to do so if the red ones are not addressed. Many times, we won't be able to address that because approach wise is very different. I do think we need some support or for some form of training (N4)*.
Theme 4: Structural barriers **(** ***implementation challenges, meso and macro level***)	**Financial challenges** *You're all here to support us, thank you for the HEALing programme, you're helping us… but we require more financial support. Especially for us with these things (pointed his foot wound), we need more support (P7)*. *Well, you see diabetes is a chronic illness. I think the major thing is the costs… let's say our dressing and we need to buy dressing materials. I think we need more subsidies on that to support our care because it's going to be long term things, as I see over the years the price has been going up and up (P8)*.	**Resources constraints** *Delivering the programme requires involvement of wound care nurses and additional time slots, some polyclinics may not have sufficient manpower resources, and time slots for service station coverage or conducive environment or appropriate venue to deliver the programme (N3)*.

## 4 Discussion

This study outlines the co‐design process for a novel, theory‐based, personalised HEALing intervention that is jointly endorsed by patients and wound care nurse practitioners to support self‐care and promote positive psychological adjustment among primary care patients living with DFU. This programme will undergo testing in a feasibility trial with flexibility for further refinement during evaluation and future implementation. Primary outcomes of the trial include feasibility (e.g., recruitment and retention rates), acceptability (e.g., participant perceptions and challenges), and implementation metrics (e.g., delivery time and resource utilisation). Secondary outcomes will evaluate HEALing's preliminary effectiveness on psychological, behavioural, knowledge and clinical measures. To our knowledge, this is the first co‐designed intervention aimed at fostering adaptive self‐care in this population.

In alignment with the UK guidance for complex intervention development frameworks [38], the iterative and dynamic co‐design process began with an exploratory phase 1 to identify key needs and determinants for intervention targets. Phase 2 utilised the EBCD approach, conducting a series of workshops with co‐designers to develop the intervention content and optimise delivery methods. In Phase 3, iterative refinements shaped the HEALing intervention pathway for a pilot investigation.

The co‐design process resulted in the development of the HEALing programme – a structured, theory‐informed intervention comprising three personalised, 30‐min face‐to‐face sessions delivered by trained wound care nurses during routine clinic visits. These nurses, already engaged in wound management and patient education, are well‐placed to integrate the intervention into existing care pathways. HEALing sessions were tailored to individual patient needs and aimed to build self‐care capacity, strengthen psychological adjustment, and empower patients through knowledge and relational support. This approach is consistent with established models of self‐management support in primary care settings, supporting individualised care [42].

The structured sequence of sessions constitutes the HEALing Pathway. This progression reflects the psychological flow of behaviour change and is explicitly aligned with core tenets of MI and SDT, particularly the promotion of autonomy, enhancement of confidence, and reinforcement of competence [24]. This pathway provides conceptual coherence for both clinical implementation and fidelity assessment and anchors intervention delivery in evidence‐based psychological mechanisms.

The EBCD approach ensured that the development of the HEALing intervention was grounded in the lived experiences of patients and the practical expertise of clinicians. This collaborative process fostered patient empowerment, strengthened therapeutic relationships, and supported shared decision‐making between patients and wound care nurses. By foregrounding patient autonomy and embedding meaningful patient and public involvement throughout, EBCD enhanced the intervention's contextual relevance, feasibility, and acceptability – maximising its potential for implementation and impact in real‐world clinical settings.

The HEALing logic model centres on patient autonomy, supported by motivational communication to enhance engagement and confidence. Collaborative agenda mapping using a visual card‐sorting tool empowers patients to prioritise care needs, identify barriers, and cocreate solutions with their wound care nurse. Grounded in SDT, HEALing promotes psychological need satisfaction and autonomous motivation, with the potential to improve self‐care and support positive psychological adjustment in patients with DFU.

HEALing empowers patients to take small and achievable steps toward adaptive and effective self‐care. Through motivational communication, patients and wound care nurse practitioners collaboratively set individual goals. Aligned with the multidisciplinary approach to DFU management, HEALing addresses patients' diverse care needs, including wound and foot care, footwear, diabetes care, timely treatment seeking, and emotional well‐being, to support optimal healing. This integration highlights HEALing's potential to enhance routine DFU care and improve patient outcomes.

Thematic analysis of co‐design workshop data revealed that the HEALing intervention was highly valued by both patient and wound care nurse participants. It was described as a personalised support and a collaborative care plan addressing psychological needs. Both groups expressed strong alignment regarding the intervention's content, delivery approach, and care pathway, as well as shared concerns about implementation challenges, including training requirements and structural barriers. This consensus underscores the intervention's potential acceptability and its potential as a collaborative model to support adaptive DFU self‐care behaviours.

During the co‐design workshops, wound nurse participants emphasised the need for additional support, particularly in training and resources, to facilitate HEALing delivery within existing operational constraints. These concerns align with previous findings, where podiatrists reported feeling unsupported in efforts to empower patients or establish partnerships [52], often due to time limitations [5, 15]. To address these challenges, the feasibility trial will integrate a dedicated HEALing appointment slot alongside routine wound care visits. Additionally, a tailored training programme will be developed to equip wound care nurses with the necessary skills and resources to deliver HEALing effectively and ensure intervention fidelity. This will include regular supervision and feedback to monitor and maintain consistent, accurate delivery of all intervention components.

Patient participants also highlighted structural barriers, particularly financial constraints, that could hinder their ability to engage in self‐care. This aligns with existing evidence showing that costs, such as those for recommended footwear, can limit adherence to care plans [5, 15]. Addressing these challenges will require collaboration between healthcare providers and social services to help patients navigate financial challenges. Strategies may include establishing partnerships with social service agencies to facilitate access to subsidies, financial assistance, or affordable therapeutic footwear, thereby reducing the financial burden on patients and supporting sustained self‐care engagement.

### 4.1 Strengths and Limitations

Our complex intervention development process has several strengths. First, by involving both patients and wound care nurses, we integrated valuable “expert” insights from lived experiences with DFU and direct patient care. This approach deepened our understanding of the condition and facilitated the cocreation of targeted, personalised intervention materials, enhancing feasibility and acceptability in real‐world settings.

Additionally, we conducted interviews with a diverse range of stakeholders, including family physicians, podiatrists, wound care nurses, and patients with varying foot conditions – post‐amputation wounds, active DFU, and post healing in remission. This comprehensive approach allowed us to identify a broad spectrum of challenges and barriers while considering adaptations for a wider diabetes population. The HEALing intervention, developed through a theory‐driven, dynamic, and adaptable EBCD process, has strong potential for scalability and sustainability in addressing diabetic foot disease risk both in Singapore and globally.

Another key strength of HEALing is its co‐design framework, guided by SDT, which fosters intrinsic motivation for sustainable, long‐term behaviour change. By integrating SDT with MI – a patient‐centred counselling approach that enhances autonomy and addresses ambivalence – HEALing moves beyond external incentives, positioning its suitability for large‐scale implementation.

However, one limitation of our study was the absence of female patient participant in the co‐design workshops. This gender imbalance is consistent with prior research at the same institution, where males accounted for nearly three‐quarters of the DFU population [13, 35]. Gender influences self‐care and illness beliefs; women with prior DFU often dislike therapeutic footwear for its impact on femininity and identity [53], which may hinder adherence and impair wound healing. The HEALing intervention, guided by MI strategies – such as the ask – offer – ask framework and collaborative action planning – aligns with evidence‐based approaches like interactive education and real‐time problem‐solving to support women's engagement in foot self‐care [54]. Given that co‐design is an iterative process, we remain committed to actively incorporating female perspectives during the pilot, feasibility, evaluation, and implementation phases to further refine and enhance the HEALing intervention.

## 5 Conclusion

This study details the co‐design and development of HEALing, a nurse‐led, clinic‐integrated intervention designed to support self‐care behaviours and emotional adjustment in patients with DFU. HEALing represents a timely and innovative advancement in DFU care, addressing the often‐overlooked psychological aspects of self‐management. Endorsed by both patients and wound care nurses, the intervention is currently undergoing feasibility evaluation in primary care.

## Author Contributions

**Xiaoli Zhu:** conceptualization, data curation, formal analysis, funding acquisition, investigation, methodology, resources, software, validation, visualization, writing – original draft preparation, writing – review and editing. **Eng Sing Lee:** funding acquisition, resources, supervision, validation, visualization, writing – review and editing. **Frederick H. F. Chan:** conceptualization, methodology, validation, visualization, writing – review and editing. **Ruoyu Yin:** validation, visualization, writing – review and editing. **Rachel W. S. Koh:** data curation, formal analysis, project administration, resources, validation, writing – review and editing. **Phoebe X. H. Lim:** data curation, project administration, resources, validation, writing – review and editing. **Carpenter Judith:** methodology, visualization, writing – review and editing. **Voon Hooi Lim:** validation, writing – review and editing. **Richard S. Y. Low:** project administration, resources, validation, writing – review and editing. **Yee Chui Chen:** project administration, resources, validation, writing – review and editing. **Yan Chen:** validation, writing – review and editing. **Xiuhong Wang:** validation, writing – review and editing. **Pei Pei Ng:** validation, writing – review and editing. **Catherine T. Y. Tan:** validation, writing – review and editing. **Sonia Tan:** validation, writing – review and editing. **Katrina Pereira:** validation, writing – review and editing. **Konstadina Griva:** conceptualization, funding acquisition, investigation, methodology, supervision, validation, visualization, writing – review and editing.

## Ethics Statement

The studies were approved by the Domain Specific Review Board Singapore (Ref No. 2021/01074 for Phase 1; Ref No. 2022/00895 for Phase 2) and Institute Review Board Nanyang Technological University (NTU IRB‐2022‐338 for Phase 1; Ref No. IRB‐2023‐335 for Phase 2).

## Consent

All study participants across all phases provided written consent.

## Conflicts of Interest

The authors declare no conflicts of interest.

## Supporting information


**Additional file 1:** Co‐design Workshop Outlines.

**Additional file 2:** Completed guidance for reporting intervention development studies in health research (GUIDED) checklist.

**Additional file 3:** Completed template for intervention description and replication (TIDieR) checklist.

**Additional file 4:** Themes and illustrative quotes from patients and wound care nurses on intervention content options identified during co‐design workshops.

**Additional file 5:** Description of card sorting task used in HEALing sessions (See Figure 3: output of Phase 2).

**Additional file 6:** Themes and illustrative quotes from patients and wound care nurses on intervention delivery options identified during co‐design workshops.

## Data Availability

Data available on request due to privacy/ethical restrictions.
