# Supplementary material for: Healing Through Empowerment and Active Listening: Experience‐Based Co‐Design of a Nurse‐Led Personalised Self‐Care Support Intervention for Primary Care Patients With Diabetic Foot Ulcers
Source: Health Expect. 2025 Aug 23;28(4):e70386. doi: 10.1111/hex.70386 (PMC12374250; doi:10.1111/hex.70386)
Supplement: Supplementary file 1 — Additional file 1: Co‐design Workshop Outlines. [file HEX-28-e70386-s002.docx]

**Healing through Empowerment and Active Listening (HEALing): Experience-Based Co-Design of a Nurse-Led Personalized Self-Care Support Intervention for Primary Care Patients with Diabetic Foot Ulcers**

**Additional file 1: Co-design Workshop Outlines**

**Workshop Preparation and Team Roles**

| **Category** | **Details** |
| --- | --- |
| **Prior to Workshops** | **Recruitment:** Ensure participant selection and invitations are completed ahead of time.  **Meeting Room Booking:** Reserve suitable rooms with necessary accessibility and comfort for participants.  **Materials Preparation:** Prepare cards, pens, sticky notes, whiteboard or flip chart, and any other materials needed for card-sorting and brainstorming activities. |
| **Workshop Team Involvement** | **Main Investigators (Facilitators):**Lead session facilitation, presenting key findings and guiding discussions. Pose open-ended questions and probe for deeper insights. Clarify participant comments to ensure accurate understanding. Facilitate and manage group discussions.  **Facilitation Tips:** Maintain a supportive, non-judgmental environment. Track time while allowing flexibility for productive conversations. |
|  | **Research Assistants:**  **First Assistant Facilitator:** Support role-playing activities and time management. Record discussions, take field notes, and ensure equitable participation.  **Second Assistant Facilitator:** Manage consent procedures with participants. Provide technical support throughout the workshop (e.g., slides, audio recording). |

**Workshop 1, 2 & 3 conducted separately with patient and wound care nurse groups: Timetable, activities and descriptions (Duration: 90 minutes each)**

| **Time** | **Activity** | **Description** |
| --- | --- | --- |
| **5 mins** | **Icebreaker** | **Patients:** Acknowledge the challenges of living with a diabetic foot ulcer (DFU), affirm their self-care expertise, and invite them to co-design HEALing to support their self-care.  **Nurses:** Acknowledge the challenges of supporting DFU care, affirm their expertise, and invite them to co-design HEALing to support patient self-care. |
| **20 mins** | **Setting the Scene: Understanding Self-Care Experiences** | **Purpose:** To explore current DFU self-care practices, challenges, and emotional barriers/enablers from the participant perspective, grounding the workshop in real experiences.  **Patients - Prompts:** “What daily actions do you take to care for your foot ulcer?” “What challenges do you face when managing foot wound care?” “How do you feel when healing is slow or the wound worsens, and what helps you keep going?  **Nurses - Prompts:** “What daily actions do your patients take to manage DFU self-care?” “What challenges do your patients face?” “What helps you support patients’ self-care, especially when they feel low?” |
| **30 mins** | **Co-Design Session: Identifying Content & Delivery Preferences** | **Purpose:** To identify what support content is most helpful for DFU self-care and explore *how, when,* and *by whom* it should be delivered for practicality and meaningfulness.  **Patients - Prompts:** “What information or support would help you feel more confident?” “How would you like to receive this support?” “When would it feel most helpful?” “Who would you feel most comfortable receiving support from?  **Nurses - Prompts:** “What topics or skills are most important for supporting DFU self-care?” “How could this support fit into your workflow?” “When in the patient journey should self-care discussions happen?” “Who should deliver this support to best motivate patients?” |
| **30 mins** | **Feedback and Refinement** | **Purpose:** To strengthen intervention content and delivery by gathering feedback and practising materials in realistic scenarios.  **Part 1 - Quick Review:** Participants review card-sorting tool and key topics.  **Patients - Prompts:** “What is helpful or confusing?”, “What would make sessions easier to follow?”, “Is anything missing or unnecessary?  **Nurses - Prompts:** “What is clear and useful?”, “What might be difficult to deliver?”, “Are there gaps or overlaps?  **Part 2 - Role Play:** Practice a short scenario using the card-sorting tool for agenda mapping, priority setting, and discussing sensitive topics (e.g., negative emotions)  **Patients - Prompts:** “How would you feel using the cards?”, “What would help you share concerns?”  **Nurses - Prompts:** “How would you introduce the cards?”, “What challenges might arise?”, “What support would help you deliver the intervention confidently?  **Debrief:** “What worked well?”, “What needs adjusting?”, “What would help maintain engagement?” |
| **5 mins** | **Closing and next steps** | Thank participants for their time and input.  Summarise key takeaways and confirm feedback will refine HEALing.  Outline next steps (e.g., joint workshops and future participation opportunities).  Reaffirm appreciation and commitment to co-creating a meaningful, practical programme. |

**Workshop 4—joint workshop: Timetable, activities and descriptions (Duration: 90 minutes)**

| **Time** | **Activity** | **Description** |
| --- | --- | --- |
| **5 mins** | **Welcome and Recap** | Thank participants for prior contributions. Reaffirm collaborative goals to finalise the HEALing intervention, ensuring it is practical, patient-centred, and feasible for clinical delivery. Share session objectives. |
| **30 mins** | **Feedback Integration & Solution Enhancement** | **Purpose:** To refine intervention content, delivery methods, and supporting materials by integrating patient and nurse feedback.  **Activities:** Review draft session structure, card-sorting tool, and materials together. Identify strengths, areas for improvement, and missing elements.  **Facilitator Prompts:** “What feedback from previous workshops stood out?” “How can we improve clarity and practicality?” “Is there anything that still feels unclear or missing?” |
| **30 mins** | **Role-Play & Finalising the Programme** | **Purpose:** To role-play the refined materials and delivery flow in realistic scenarios, addressing sensitive topics (e.g., managing worries) and using the card-sorting tool for agenda mapping.  **Activities: O**ne acts as nurse, one as patient, others observe and note feedback. Focus on introducing the cards, exploring self-care priorities, and addressing emotional concerns.  **Facilitator Prompts:** “How did it feel to introduce and use the cards?” “What helped or hindered discussion?” “What support or training would help delivery feel comfortable and effective?” |
| **20 mins** | **Reflection and Next Steps** | **Purpose:** To consolidate learning, confirm adjustments, and plan for pilot intervention.  **Activities:** Group discussion on key takeaways from role-play and review. Identify any final adjustments needed for clarity, flow, or feasibility. Discuss training needs and support for nurses, and participation expectations for patients.  **Facilitator Prompts:** “What worked well today, and what needs refining before implementation?” “For nurses: Would you be interested in training to facilitate HEALing? Why or why not?” “For patients: Would you be interested in participating in HEALing? Why or why not?” “What support will help us sustain this programme in practice?” |
| **5 mins** | **Closing and Appreciation** | Thank participants for their time and insights, reaffirming their essential role in shaping HEALing. Share next steps for pilot evaluation and future collaboration. |
