# Supplementary material for: Healing Through Empowerment and Active Listening: Experience‐Based Co‐Design of a Nurse‐Led Personalised Self‐Care Support Intervention for Primary Care Patients With Diabetic Foot Ulcers
Source: Health Expect. 2025 Aug 23;28(4):e70386. doi: 10.1111/hex.70386 (PMC12374250; doi:10.1111/hex.70386)
Supplement: Supplementary file 2 — Additional file 2: Completed guidance for reporting intervention development studies in health research (GUIDED) checklist. [file HEX-28-e70386-s003.docx]

**Healing through Empowerment and Active Listening (HEALing): Experience-Based Co-Design of a Nurse-Led Personalized Self-Care Support Intervention for Primary Care Patients with Diabetic Foot Ulcers**

**Additional File 2 Completed guidance for reporting intervention development studies in health research (GUIDED) checklist**

| Item description | Page in manuscript where item is located | Others* |
| --- | --- | --- |
| 1. Report the context for which the intervention was developed. | 4-5 |  |
| 2. Report the purpose of the intervention development process. | 6-7 |  |
| 3. Report the target population for the intervention development process. | 7-10 |  |
| 4. Report how any published intervention development approach contributed to the development process | 11-12 |  |
| 5. Report how evidence from different sources informed the intervention development process. | 11-12 | 1,2,3,4 |
| 6. Report how/if published theory informed the intervention development process. | 5-6, 11-12 | 5,6,7,8 |
| 7. Report any use of components from an existing intervention in the current intervention development process. | Table 1,2 |  |
| 8. Report any guiding principles, people or factors that were prioritised when making decisions during the intervention development process. | 14-15 |  |
| 9. Report how stakeholders contributed to the intervention development process. | 14-17 |  |
| 10. Report how the intervention changed in content and format from the start of the intervention development process | 15 |  |
| 11. Report any changes to interventions required or likely to be required for subgroups. | NA |  |
| 12. Report important uncertainties at the end of the intervention development process. | NA |  |
| 13. Follow TIDieR guidance when describing the developed intervention. | 14-17 |  |
| 14. Report the intervention development process in an open access format. | 14-17 |  |
| *References   1. O’Cathain A, Croot L, Duncan E, Rousseau N, Sworn K, Turner KM, et al. Guidance on how to develop complex interventions to improve health and healthcare. BMJ Open [Internet]. 2019 Aug 1 [cited 2023 Jan 27];9(8). Available from: /pmc/articles/PMC6701588/ 2. Donetto S, Pierri P, Tsianakas V, Robert G. Experience-based Co-design and Healthcare Improvement: Realizing Participatory Design in the Public Sector. The Design Journal [Internet]. 2015 [cited 2025 Jan 21];18(2):227–48. Available from: https://www.tandfonline.com/doi/abs/10.2752/175630615X14212498964312 3. Bielinska AM, Archer S, Darzi A, Urch C. Co-designing an intervention to increase uptake of advance care planning in later life following emergency hospitalisation: a research protocol using accelerated experience-based co-design (AEBCD) and the behaviour change wheel (BCW). BMJ Open [Internet]. 2022 May 1 [cited 2025 Jan 17];12(5):e055347. Available from: https://bmjopen.bmj.com/content/12/5/e055347 4. McAllister S, Simpson A, Tsianakas V, Canham N, De Meo V, Stone C, et al. Developing a theory-informed complex intervention to improve nurse–patient therapeutic engagement employing Experience-based Co-design and the Behaviour Change Wheel: an acute mental health ward case study. BMJ Open [Internet]. 2021 May 1 [cited 2025 Jan 17];11(5):e047114. Available from: <https://bmjopen.bmj.com/content/11/5/e047114> 5. Deci EL, Ryan RM. The “What” and “Why” of Goal Pursuits: Human Needs and the Self-Determination of Behavior. Psychol Inq. 2000;11(4):227–68 6. Deci EL, Ryan RM. Facilitating Optimal Motivation and Psychological Well-Being Across Life’s Domains. 2008; 7. Markland D, Ryan RM, Tobin VJ, Rollnick S. MARKLAND ET AL. MOTIVATIONAL INTERVIEWING MOTIVATIONAL INTERVIEWING AND SELF-DETERMINATION THEORY. J Soc Clin Psychol. 2005;24(6):811–31. 8. Miller WR, Rose GS. Toward a Theory of Motivational Interviewing. American Psychologist. 2009 Sep;64(6):527–37. | | |

Adapted from: Duncan, E., et al., *Guidance for reporting intervention development studies in health research (GUIDED): an evidence-based consensus study.* BMJ Open, 2020. **10** (4): p. e033516.
