# Supplementary material for: Healing Through Empowerment and Active Listening: Experience‐Based Co‐Design of a Nurse‐Led Personalised Self‐Care Support Intervention for Primary Care Patients With Diabetic Foot Ulcers
Source: Health Expect. 2025 Aug 23;28(4):e70386. doi: 10.1111/hex.70386 (PMC12374250; doi:10.1111/hex.70386)
Supplement: Supplementary file 3 — Additional file 3: Completed template for intervention description and replication (TIDieR) checklist. [file HEX-28-e70386-s001.docx]

**Healing through Empowerment and Active Listening (HEALing): Experience-Based Co-Design of a Nurse-Led Personalized Self-Care Support Intervention for Primary Care Patients with Diabetic Foot Ulcers**

**Additional File 3 Completed template for intervention description and replication (TIDieR) checklist**

| **Item No.** | **Description** | **Item** | **Reported in this paper (Y/N)** |
| --- | --- | --- | --- |
| 1 | Brief name | Provide the name or a phrase that describes the intervention | Page 7 |
| 2 | Why | Describe any rationale, theory, or goal of the elements essential to the intervention | Page 4-7 |
| 3 | What | Materials: Describe any physical or informational materials used in the intervention, including those provided to participants or used in intervention delivery or in training of intervention providers. Provide information on where the materials can be accessed (such as online appendix, URL) | Page 15-16  (Physical cards) |
| 4 |  | Procedures: Describe each of the procedures, activities, and/or processes used in the intervention, including any enabling or support activities | Page 14-17 |
| 5 | Who provided | For each category of intervention provider (such as psychologist, nursing assistant), describe their expertise, background, and any specific training given | Page 9 |
| 6 | How | Describe the modes of delivery (such as face to face or by some other mechanism, such as internet or telephone) of the intervention and whether it was provided individually or in a group | Page 16-17 |
| 7 | Where | Describe the type(s) of location(s) where the intervention occurred, including any necessary infrastructure or relevant features | Page 16-17 |
| 8 | When and how much | Describe the number of times the intervention was delivered and over what period of time including the number of sessions, their schedule, and their duration, intensity, or dose | Page 16-17 |
| 9 | Tailoring | If the intervention was planned to be personalised, titrated or adapted, then describe what, why, when, and how | Page 15-18 |
| 10 | Modifications | If the intervention was modified during the course of the study, describe the changes (what, why, when, and how) | Page 15-16 |
| 11 | How well | Planned: If intervention adherence or fidelity was assessed, describe how and by whom, and if any strategies were used to maintain or improve fidelity, describe them | NA (this will be addressed in pilot trial) |
| 12 |  | Actual: If intervention adherence or fidelity was assessed, describe the extent to which the intervention was delivered as planned |  |

Adapted from: Hoffmann, T.C., et al., *Better reporting of interventions: template for intervention description and replication (TIDieR) checklist and guide.* BMJ : British Medical Journal, 2014. **348**: p. g1687
