# Supplementary material for: Healing Through Empowerment and Active Listening: Experience‐Based Co‐Design of a Nurse‐Led Personalised Self‐Care Support Intervention for Primary Care Patients With Diabetic Foot Ulcers
Source: Health Expect. 2025 Aug 23;28(4):e70386. doi: 10.1111/hex.70386 (PMC12374250; doi:10.1111/hex.70386)
Supplement: Supplementary file 4 — Additional file 4: Themes and illustrative quotes from patients and wound care nurses on intervention content options identified during co‐design workshops. [file HEX-28-e70386-s004.docx]

**Healing through Empowerment and Active Listening (HEALing): Experience-Based Co-Design of a Nurse-Led Personalized Self-Care Support Intervention for Primary Care Patients with Diabetic Foot Ulcers**

**Additional file 4. Themes and illustrative quotes from patients and wound care nurses on intervention content options identified during co-design workshops**

| **Themes (content options/topics)** | Patients and wound care nurses described their priorities for self-care knowledge and skills, care engagement, and psychological concerns through sharing personal experiences and reflections. These discussions highlighted the daily realities and challenges of living with a diabetic foot ulcer, emphasizing the need for practical and emotional support. Five non-ranking overarching topic categories emerged:   1. **Wound care**: dressing maintenance; recognising wound deterioration 2. **Foot care and footwear**: foot skin care; routine foot inspections; wearing appropriate footwear 3. **Diabetes care**: glycaemic control; dietary adherence; medication adherence 4. **Treatment seeking:** timely treatment/appointment attendance 5. **My concerns and worries**: fears about the wound; family relationships; how do I look to others |
| --- | --- |
| **Perceived by patients (illustrative quotes)** | *Based on my condition when I lost my toes, so self-care is very important yes, self-care is very important. The first thing you continue to control your diabetes. In addition, then you check your foot and footwear, especially after you have your shower and then everything make sure it’s clean. You need to take care of your wound dressing, take your medicine and attend your treatment appointments. I mean if you miss any of them, you’re just putting yourself back into certain risks. (P3,* ***topics a,b,c,d****)*  *Diet control for diabetes care, then foot self-care, moisturizer foot dry skin, self-checking foot daily, footwear … everyone must do all these every day. Because I already lost a toe, attending appointment is important. (P4,* ***topics a,b,c****)*  *I think the first thing is, we must know what causes us to get a new wound? How did the new wound come from? How to prevent them? So diabetes, foot and shoes, and seeing doctors are needed (P1,* ***topics b,c,d****)*  *Although we have a diabetes team to take care of. Now, from this session, I feel that this diabetes control is good for us also to talk to you because it’s all linked. Always wear shoes outdoor, and If anything touch your feet, poke your feet, don’t take the risk. Go straight to the doctor.” Timely treatment is crucial. I feel we need to know that we must wear some form of footwear in house as we have diabetes, our nerves are also going to get affected. One of the most important tasks is following diet and medication advice. I cannot follow these, that’s why HbA1c is my problem. (P3,* ***topics b,c,d****)*  *We need to know the root cause of diabetes, like our diet! This is something that we need to learn first. It’s very important especially we have a wound. Wound care, foot care and early attendance to see doctors also important. (P4,* ***topics a,b,c,d****)*  *When I go to toilet and bathe, wound dressing hits the water, and gets wet. So it’s very difficult for us to maintain wound dressing. (P5,* ***topic a****)*  *I mean, foot ulcer is one thing which you can never predict. So what are the cause of this ulcer becoming worse? We can do some preventive measures besides just monitoring you know?  I feel that kind of self-care knowledge and skills on how to recognize infection and manage wound that we want to understand more. (P8,* ***topics a****)*  *We also need to learn to change wound dressing, wearing the off-loading shoe, checking feet and shoes. All these things are part of self-care, yeah important part of self-care... The nurse got to do also. If we inspect anything wrong, we must tell the nurse immediately. So we collaborate with the nurse. The nurses must help us. (P9,* ***topics a,b,d****)*  *Wearing dressing taking bath is very difficult to maintain dressing dry. (P7,* ***topics a****)*  *I guess we need to accept our situations like worry about what people think and burden to family. We need to learn how to move forward. (P4,* ***topic e****)*  *There’ll be a lot of anxiety and fear. You’ll start thinking and start worrying, “where does this lead to? … If you’re going to let what other people say stress you out, everything will start falling apart. Because the minute you lose confidence in yourself, everything will fall apart because you give up. So I think what is important is to accept like that he (P4) said, we need to learn how to accept it and move forward positively. (P3,* ***topic e****)* |
| **Perceived by wound care nurses (illustrative quotes)** | *I think from the very start, they probably don’t even know what’s going on with their foot. So it’s the understanding of the wound and wound care, foot care and footwear, diabetes care, and regular dressing appointment. I believe these are what most of us encountered during our care (N5,* ***topics a,b,c,d****)*  *I think all these, care of wound, foot and footwear, diabetes care, and treatment seeking, as well as care of negative mood, are definitely very significant and have direct impact on managing DFU itself, so the relevancy to patients and us, is definitely there. Okay, from the perspective of just what this is about right, it all has direct link to self-care outcomes and delivery. Whether it is important or not each aspect has its significance. (N2,* ***topics a,b,c,d,e****)*  *I think all the points are important and relevant to improve patient care. But the challenge is the emotional stress that patients experience especially when they come back from hospital after first amputation. It is challenging. (N3,* ***topics a,b,c,d,e****)*  *If my patient works as a cleaner, I need to tell them, “when you work in area involving water, then you need to do some precautions to keep your dressing dry. If you work as a security guard, after you finish your patrols and rounds right, you should rest your legs or just to exercise your legs to reduce the pressure over your toes. Also need to ask them to seek treatment immediately if the wound appears abnormal. Yes, all these are very important. (N3,* ***topics a,b****)*  *I think the emotional distress for patients is really a challenge in managing diabetic foot ulcer. Like progression of the disease, and the long healing process of the patients. So I think the challenging part for all the nurses in wound care dressing will be like patient support honestly. (N4,* ***topic e****)*  *They feel stressed and guilty and put burden to the family. (N1,* ***topic e****)* |
