# Supplementary material for: Healing Through Empowerment and Active Listening: Experience‐Based Co‐Design of a Nurse‐Led Personalised Self‐Care Support Intervention for Primary Care Patients With Diabetic Foot Ulcers
Source: Health Expect. 2025 Aug 23;28(4):e70386. doi: 10.1111/hex.70386 (PMC12374250; doi:10.1111/hex.70386)
Supplement: Supplementary file 5 — Additional file 5: Description of card sorting task used in HEALing sessions (See Figure 3: output of Phase 2). [file HEX-28-e70386-s005.docx]

**Healing through Empowerment and Active Listening (HEALing): Experience-Based Co-Design of a Nurse-Led Personalized Self-Care Support Intervention for Primary Care Patients with Diabetic Foot Ulcers**

**Additional file 5. Description of card sorting task used in HEALing sessions (See Figure 3: output of Phase 2)**

- All cards are double sided, ie., one side presenting words and the other side showing picture of the content.
- At the start of the first HEALing session, patients were shown two charts with two large circles:
  - *“Managed well”*
  - *“Managed not so well or have most questions”*

*
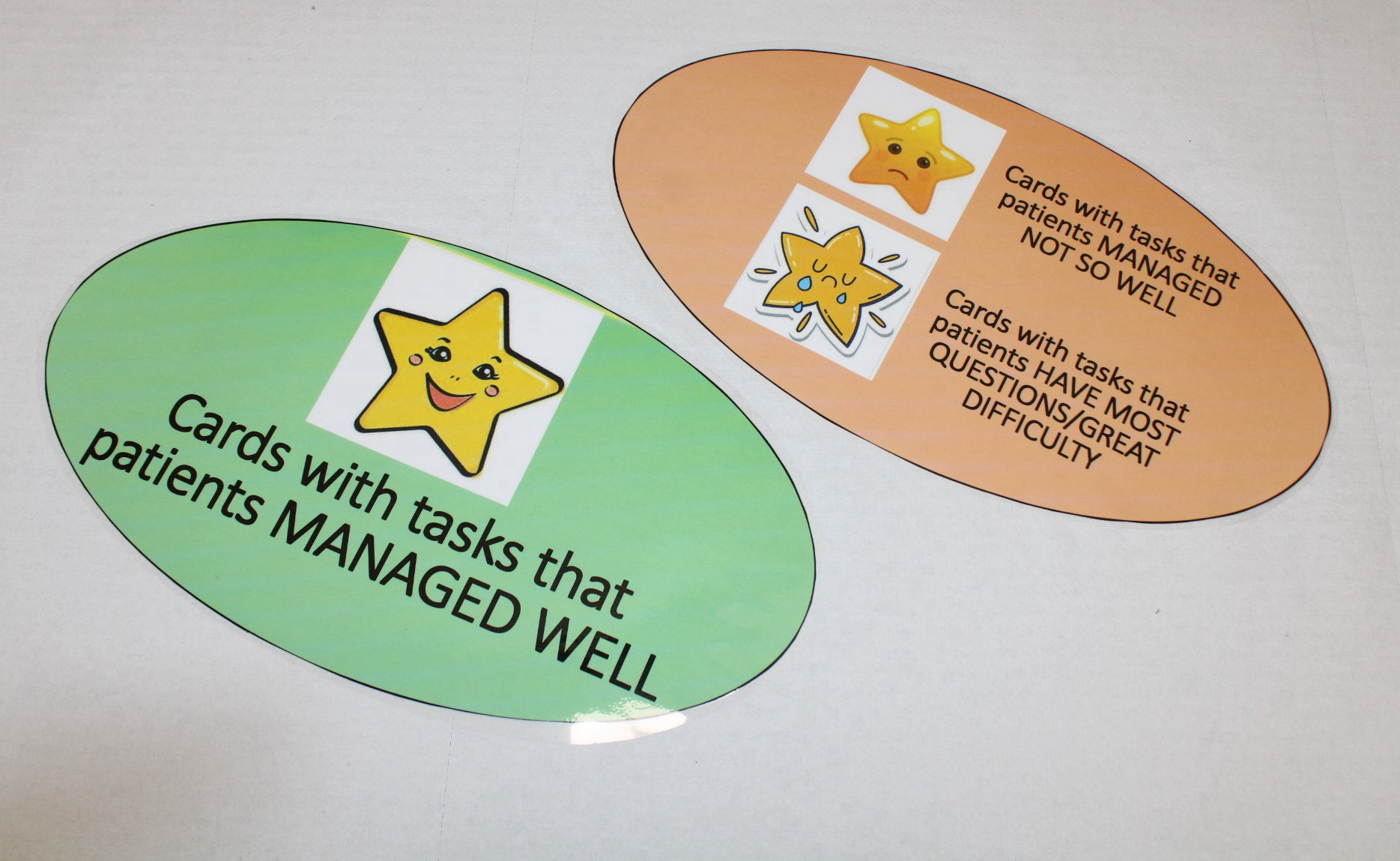
*

- Patients were provided with **colour-coded cards** representing the twelve subtopics reflecting essential clinical and emotional self-care needs.


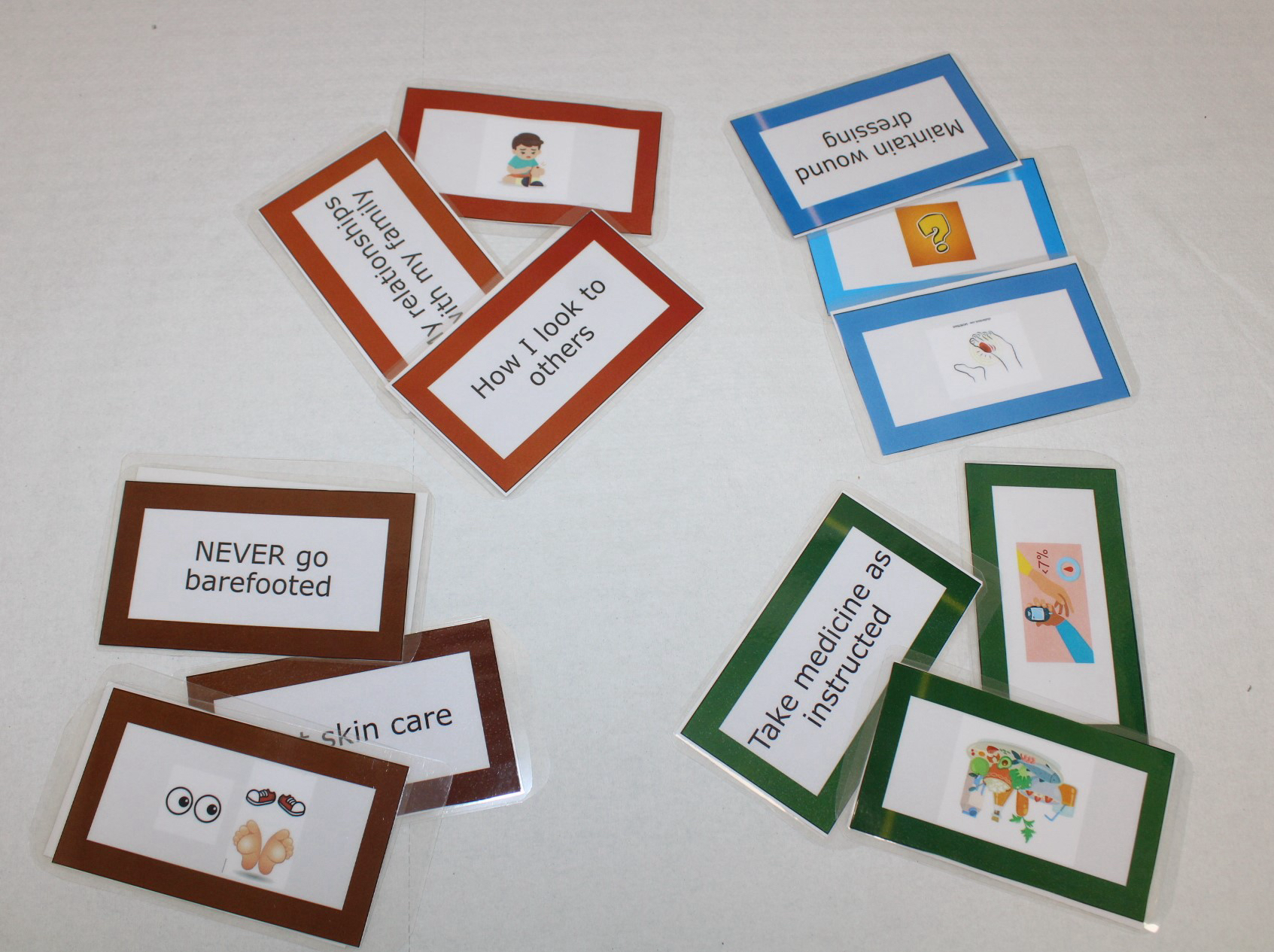


- Patients were asked to **sort the cards** into the two categories:
  - Tasks they felt they *“managed well”.*
  - Tasks they felt they *“managed not so well or have most questions”.*


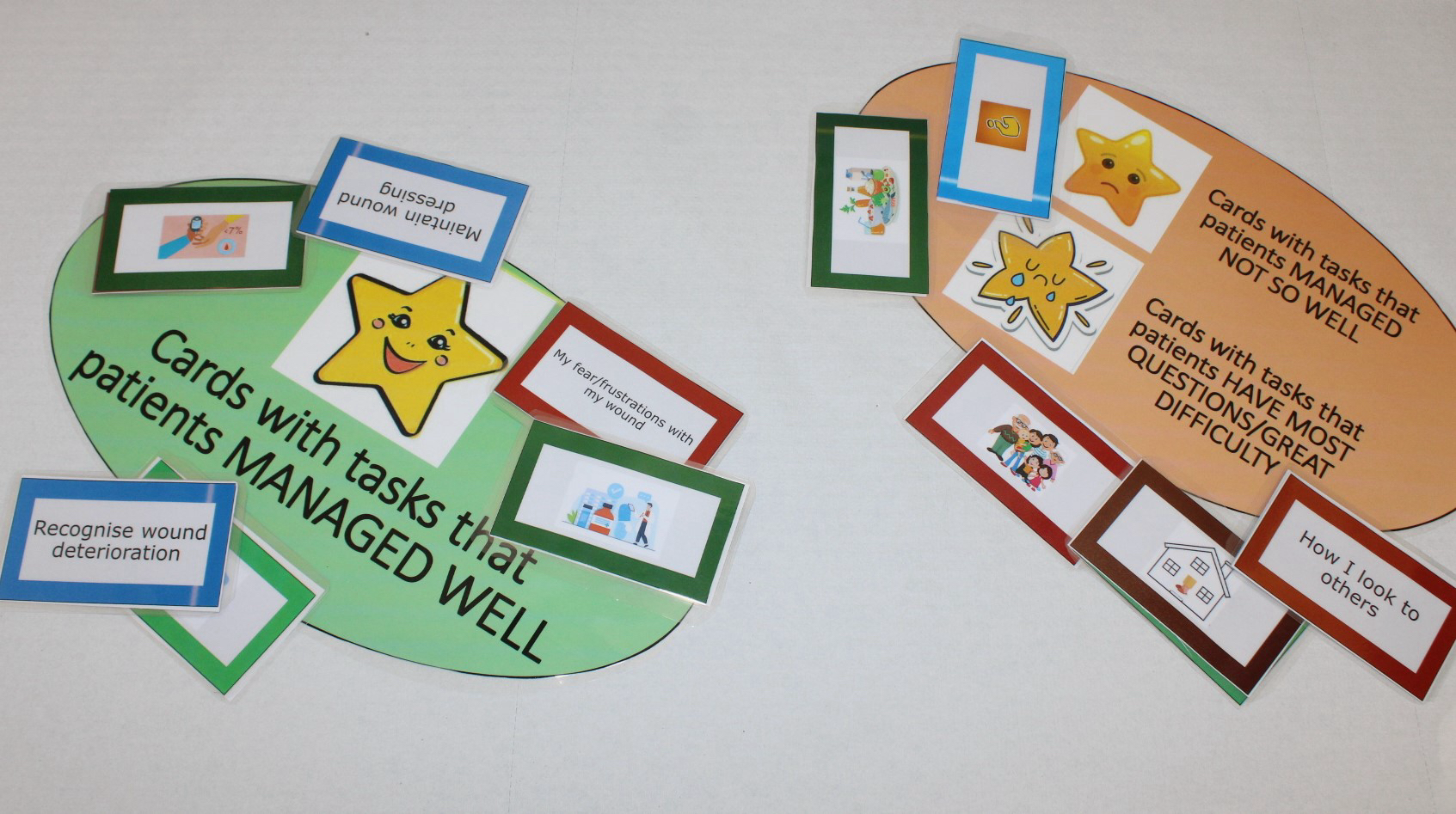


- This process:
  - Encouraged patients to **reflect on their self-care practices.**
  - Enabled wound care nurses to **affirm patients’ strengths and efforts** before exploring areas needing additional support.
- Using an **ask-offer-ask approach**, wound care nurses facilitated discussions that:
  - Acknowledged patient achievements.
  - Collaboratively identified **priority self-care needs** for targeted support.
- The card sorting task **guided agenda setting** for the HEALing intervention by:
  - Ensuring sessions were **tailored to individual needs.**
  - Promoting **patient autonomy and active engagement** in care planning.
