# Supplementary material for: Healing Through Empowerment and Active Listening: Experience‐Based Co‐Design of a Nurse‐Led Personalised Self‐Care Support Intervention for Primary Care Patients With Diabetic Foot Ulcers
Source: Health Expect. 2025 Aug 23;28(4):e70386. doi: 10.1111/hex.70386 (PMC12374250; doi:10.1111/hex.70386)
Supplement: Supplementary file 6 — Additional file 6: Themes and illustrative quotes from patients and wound care nurses on intervention delivery options identified during co‐design workshops. [file HEX-28-e70386-s006.docx]

**Healing through Empowerment and Active Listening (HEALing): Experience-Based Co-Design of a Nurse-Led Personalized Self-Care Support Intervention for Primary Care Patients with Diabetic Foot Ulcers**

**Additional file 6. Themes and illustrative quotes from patients and wound care nurses on intervention delivery options identified during co-design workshops**

| **Themes (delivery options)** | Patients and wound care nurses described preferences for *How, When, By Whom* the intervention should be delivered, using the card-sorting tool to facilitate discussion. Key delivery preferences included: 1) individualized one-to-one sessions ***(How),*** 2) integration into routine clinic visits ***(When),*** and 3) delivery by the same wound care nurse to ensure continuity and trust ***(Who).*** |
| --- | --- |
| **Perceived by patients (illustrative quotes)** | *I like the 12 cards (card-sorting task). The card-sorting game with 12 cards is a very good thing, if it is done one to one makes it even better as we can open up and share more for help. (P3;* ***How****)*  *The contents on the cards -- all has direct link to managing DFU itself. Individualized session is good as I can open up and share my feelings and my problems. However, if I’m in the room, with quite a number of people, then I kind of restrict myself and reserve myself from talking. If I say this, what the other person will think about me. (P3,* ***How****)*  *It’s better the same nurse, because they’ve been with us seeing our wound every other day, so the rapport is already there. But the nurses should have some allocated time to do that [intervention] with us after wound dressing. (P7,* ***How, Who, When****)*  *Patients must see the same nurse because of therapeutic patient-nurse relationship.* *(P8,* ***Who****)*  *I think it should be one-to-one during clinic visit because of therapeutic patient-nurse relationship. I can open up and tell them my problem, without repeating the whole story again.* *(P1,* ***Who, How, When****)*  *Like for instance if I’m seeing the same nurse most of the time, and she connects with me. The minute I walk in, the nurse says, “hey you’re here!”, starts with me right with the card sorting task. As she connects with me better, I tend to listen more. You get where I’m coming from, the psychological side of the whole* thing? Because patients sometimes need somebody to connect with me. *(P2,* ***Who, How****)* |
| **Perceived by wound care nurses (illustrative quotes)** | *The card-sorting game helps us identify what the patient wants to know and do, especially if it is scheduled after dressing session. I think this will help us to break the barrier or overcome the difficulties between the wound nurses and the patients (N3,* ***How, When****)*  *“One-to-one session during patient’s wound visit helps us to better understand patient’s unique needs, build up rapport and providing targeted personalized care” (N5,* ***How, When****)*  *Nurses often have the most contact time with patients, placing them in a prime position to make a significant difference through effective counselling and education. (N2,* ***Who****)*  *One-to-one card-sorting task gives patients autonomy to choose and decide their care with support from nurses. They know what they manage well, what they had most problems with and wanted to seek support from us. (N3,* ***How****)*  *We’ve been seeing patients once 3 times or even daily dressing, so we roughly know how’s the patients’ progress. Beginning with card-sorting task not only focusing on the challenges the patients faced but also the success part they have been doing well. So at least this part will help to motivate patients of how far they’ve come through all these things. (N4,* ***How****)*  *Regarding the card right, It’s a bit wordy. I was thinking, if possible, add a small picture. It's easier with visual. Or medicine just put picture of pills. (N6,* ***How***)  *So I think using card-sorting task- like recall what they have done well, what they have not. And we often miss to address the emotion part because the time constraints. Those are important areas we need to explore after wound dressing… that’s what we need during the 30 minutes one to one session with a patient. (N5,* ***How,*** ***When****)*  *I think the techniques and the timeslots-resources that we need to speak to patients really matter. So that we can drive that 30 minutes after dressing to be very effective. … but they may choose more than 1 card [more topics] for discussion, maybe with just 1 card 1 session after dressing that fills up the additional 30 minutes. So then how we follow up the pace to other cards [topics] and then how we recall it back as patients also need time to process, perhaps one to two weeks interval. (N2,* ***How, When****)*  *Good to have multiple sessions. Emotional challenges are very personal. Perhaps tackling this issue should be considered in the following session not just on the very first session where establishing rapport between patient and clinician is definitely very important during 1^st^ session. Therefore, once we get a better understanding and rapport, it’s easier for them to open-up. (N2,* ***How****)* |
